# Preface

**Published:** 1828-04

**Authors:** 


### PREFACE.

In the prospectus accompanying the first number of this volume, the Editor apprised those to whom it was sent, that being on a similar plan to the Western Medical and Physical Journal, it might serve as a continuation of that work, to all who chose to receive it as such. At that time it was uncertain whether the journal to which he refers, would be continued; and he promised to send to new subscribers a different title page for this volume, from that with which he would furnish those who had taken that work. Its discontinuance, however, renders such a measure unnecessary; and the Editor sends forth this volume (its title substantially the same with that of its predecessor) as the second volume of the work which he projected in 1827.

The Editor has devoted more time to this, than the first volume; but misfortunes, which he has already made known to his subscribers, have prevented his bestowing upon it as much labour as he hopes to exert on the work in future.

In originally fixing on the plan of monthly numbers for his work, the Editor was governed, chiefly, by the opinion, that frequent publications would be most aceptable to the profession, as communicating earlier intelligence. He was not aware, however, of the greater labour and expense attendant on frequent publications; of the difficulty of finding room in a number consisting of fifty pages only, for extended reviews; or of the increased chances of loss by the mail, from sending off twelve numbers a year, instead of four. He has determined, therefore, after consulting some of his valued friends and correspondents, to publish the work, hereafter, in quarterly numbers, of 150 pages each. The first number of the third volume, will be ready for delivery, by the end of June. It will be printed on paper and put up in a style, decidedly superior to the preceding volumes, over which the Editor flatters himself, it will rise still further in the character of its contents.

The first article of each of the four numbers of the next volume, will be devoted to the important subject of Medical Education, particularly in the Western states; a subject to which the Editor has devoted some attention, and which he hopes to treat in such manner as may contribute something to the advancement of the profession, in dignity and usefulness.

The first number will also contain a practical paper on Auscultation, with an engraving of the Stethoscope; an instrument of diagnosis, with the use of which every physician should be familiar.

The Editor has made arrangements to obtain, at an early period after their publication, all the most important systematic and monographic treatises, the substance of which he proposes to give in the form of analytical reviews; similar to those of De Candolle's Essay on the Medical Properties and Affinities of Plants; Abernethy's Lectures on Anatomy, Physiology and Surgery; and Civiale's Treatise on Lithontrity, contained in the present volume. Among the subjects which in the prosecution of this plan he intends, at an early period, to present, *in extenso*, are the Doctrines and Practice of Broussais.

